# EGF Contributes to Hypertrophy of Human Ligamentum Flavum via the TGF-β1/Smad3 Signaling Pathway

**DOI:** 10.7150/ijms.76077

**Published:** 2022-08-29

**Authors:** Kaifan Yang, Yanlin Chen, Xin Xiang, Yanling Lin, Chengshuo Fei, Zesen Chen, Zhongming Lai, Yongpeng Yu, Ruiqian Tan, Jiale Dong, Junxiong Zhang, Peng Li, Liang Wang, Zhongmin Zhang

**Affiliations:** 1 Division of Spine Surgery, Department of Orthopaedics, Nanfang Hospital, Southern Medical University, Guangzhou, China; 2 The First School of Clinical Medicine, Southern Medical University, Guangzhou, China; 3 Department of Radiation Oncology, Nanfang Hospital, Southern Medical University, Guangzhou, China; 4 Department of Orthopedics, The Third Affiliated Hospital, Southern Medical University, Academy of Orthopedics, Guangzhou, China

**Keywords:** ligamentum flavum, hypertrophy, EGF, TGF-β1, Smad3

## Abstract

**Background:** The most common spinal disorder in elderly is lumbar spinal canal stenosis (LSCS). Previous studies showed that ligamentum flavum hypertrophy (LFH) with fibrosis as the main pathological change is one of the pathogenic factors leading to LSCS. Epidermal Growth Factor (EGF) is known to have an intimate relationship with fibrosis in various tissues. Nevertheless, currently, there are few studies regarding EGF in LFH. The effect of EGF on the development of LFH is unknown, and the underlying pathomechanism remains unclear. In this study, we investigated the role of EGF in LFH and its potential molecular mechanism.

**Methods:** First, the expression levels of EGF, phosphorylation of EGF receptor (pEGFR), Transforming growth factor-β1 (TGF-β1), Phosphorylated Smad3 (pSmad3), collagen I and collagen III were examined via immunohistochemistry and Western blot in LF tissues from patients with LSCS or Non-LSCS. Second, primary LF cells were isolated from adults with normal LF thickness and were cultured with different concentrations of exogenous EGF with or without erlotinib/TGF-β1-neutralizing antibody.

**Results:** The results showed that EGF, pEGFR, TGF-β1, pSmad3, collagen I and collagen III protein expression in the LSCS group was significantly higher than that in the Non-LSCS group. Meanwhile, pEGFR, TGF-β1, pSmad3, collagen I and collagen III protein expression was significantly enhanced in LF cells after exogenous EGF exposure, which can be notably blocked by erlotinib. In addition, pSmad3, collagen I and collagen III protein expression was blocked by TGF-β1-neutralizing antibody.

**Conclusions:** EGF promotes the synthesis of collagen I and collagen III via the TGF-β1/Smad3 signaling pathway, which eventually contributes to LFH.

## Introduction

Lumbar spinal canal stenosis (LSCS) is more common in the elderly, usually lead to pain in the lower back and legs, numbness of lower limbs, and intermittent claudication 1. Lumbar disc herniation, facet joint degeneration, ligamentum flavum hypertrophy (LFH) and other pathogenic factors lead to the development of lumbar spinal canal stenosis (LSCS). Among them, ligamentum flavum hypertrophy is the most important pathogenic factor, and it is highly related to the pathogenesis of lumbar spinal canal stenosis (LSCS) 2, 3. The normal ligamentum flavum is an elastic structure composed of elastic fibers (80%) and collagen fibers (20%) 4-6. However, with the occurrence of ligamentum flavum hypertrophy, it shows the loss of elastic fibers and the increase of collagen fibers, suggesting changes in fibrosis. According to previous reports, the increased collagen fibers are mainly collagen I and collagen III 7. In other words, the main pathology of ligamentum flavum hypertrophy is fibrosis 8.

Epidermal growth factor (EGF) is a single-chain polypeptide with a molecular weight of approximately 6 kDa. In both human fibroblasts and various mammalian tissues, EGF receptors can be detected 9, 10. EGF triggers a number of biological responses, including cell proliferation and differentiation 11. EGF significantly increased expression of type I collagen in cultured primary cardiac fibroblasts 12. EGF involved in the pathology of airway fibrosis by induction of IL-8 from airway epithelium, subsequently causing lung fibroblasts proliferation and migration 13. EGF receptor (EGFR) antagonist could attenuate liver fibrosis. Specifically blocking EGF could attenuate bile duct ligation (BDL)-induced liver fibrosis 14. Previous studies have shown that EGF is associated with tissue fibrosis in many organs, including the heart, lungs, and liver.

The Transforming growth factor-β (TGF-β) family plays a crucial role in the pathogenesis of various diseases, such as fibrosis in many organs and tissues 15-17. There are three isoforms of TGF-β in mammals, including TGF-β1, TGF-β2, and TGF-β3 18. Although all three isoforms exhibit similar biological activities, previous studies suggest that tissue fibrosis is mainly associated with the TGF-β1 isoform 19, 20.Likewise, TGF-β1 expression was observed in hypertrophic ligamentum flavum. Current studies have shown that TGF-β1 plays an important role in ligamentum flavum hypertrophy (fibrosis). However, the underlying mechanism of the association between TGF-β1 and ligamentum flavum hypertrophy has not been clearly elucidated. As a downstream pathway molecule of TGF-β1, Smad family has been confirmed to be involved in regulating the physiological activities of various cells together with TGF-β1 21. The interaction of TGF-β with its receptors plays different functional roles in distinct injuries and is influenced by activated Smad3 22, 23. Activated Smad3 and Smad4 in the nucleus drive the expression of TGF-β1-targeted genes. TGF-β1 can also initiate profibrotic processes independently of Smads through transactivation of the EGF receptor (EGFR) 24, 25.

However, it is unknown whether EGF is activated in the ligamentum flavum or whether it is involved in ligamentum flavum hypertrophy. Based on the above discussion, we hypothesized that EGF may promote the production of collagen fibers that contribute to ligamentum flavum hypertrophy (LFH) by activating the TGF-β1 signaling pathway. Our study aims to discover the role of EGF in ligamentum flavum hypertrophy and reveal its possible mechanisms.

## Materials and Methods

### Ligamentum flavum tissue samples

Thirty LSCS patients(18 men and 12 women, mean age: 58.35 years) with severe LF hypertrophy and twenty Lumbar disc herniation(LDH) patients (8 males and 12 females, mean age 55.20 years) without obvious LF hypertrophy were included in this study. The ligamentum flavum tissue of the L4/5 segment was taken and washed with physiological saline for 3 times. A portion of each sample was fixed in 4% paraformaldehyde for hematoxylin and eosin (H&E) staining, Masson trichrome staining, and immunohistochemical analysis. The remaining samples were immediately stored in liquid nitrogen for subsequent Western blot analysis.

Each patient signed a written informed consent, and the Institutional Ethics Review Board of Nanfang Hospital of Southern Medical University approved this study.

### Measurement of LF thickness

Preoperative magnetic resonance images(MRI, Philips, Amsterdam, Netherlands) were collected from all 50 patients, two parallel lines were drawn along the direction of the ligamentum flavum on axial T2-weighted images through the facet joint, and the maximum distance between the dural side and the dorsal side was chosen, using PACS(Picture Archiving and Communication Systems) workstations (an image analyzing system, Nanfang Hospital of Southern Medical University, Guangzhou, China) to measure the maximum thickness of the LF. Each ligament flavum was measured three times, and the average value was taken as the final LF thickness 26, 27.

### Fibrosis evaluation of LF

Paraffin-embedded tissues were cut into 4 µm thick sections using a paraffin microtome (RM2125 RTS, Leica, Wetzlar, Germany). The sections were stained using H&E staining and Masson trichrome staining kits (JianChen, Nanjing, China). H&E staining was used to analyze the morphologic changes. The fibrous lesion was evaluated by Masson trichrome staining. Similar to previous research^7^, fibrosis score was evaluated according to the proportion of collagen fibers in the entire area: grade 0, ≤20% of the area; grade 1, 21%-40% of the area; grade 2, 41%-60% of the area; grade 3, 61%-80% of the area; and grade 4, >81% of the area.

### Immunohistochemical analyses

LF tissue sections were incubated in 3% H_2_O_2_ to quench endogenous peroxidase activity. After antigen retrieval and blocking treatment, primary antibodies against EGF (1: 200, Abcam, Cambridge, UK), phosphorylated EGFR (pEGFR) (1: 100, Cell Signaling Technology, Danvers, MA), TGF-β1(1: 100, Abcam), phosphorylated Smad3 (pSmad3) (1: 200, Cell Signaling Technology, Danvers, MA), collagen I (1: 100, Abcam), and collagen III (1: 100, Abcam) were applied overnight at 4°C. After being washed with PBS, tissue sections were incubated with a goat-anti-rabbit horseradish peroxidase-conjugated secondary antibody (1: 100, RayBiotech, Beijing, China) for 1 h at room temperature, and the immunostain signal was developed using 3, 3'-diaminobenzidine (DAB, ZSGB-Bio, Beijing, China) 28. Then, the quantitative analysis of immunohistochemical staining was conducted. The H-score was assigned to each immunohistochemistry image according to the proportion of staining area in the entire area: negative (score 0-1), positive (score 2-4), and strongly positive (score 5-9).

### Culture and identification of LF cells

The cells were isolated from the LF tissues of normal thickness. After washing in physiological saline 3 times, the specimens were minced with microdissection scissors under aseptic conditions and digested for 1.5 h at 37°C using 0.2% type I collagenase (Sigma-Aldrich, St. Louis, MO). The cells were then filtered through a sterile nylon mesh filter (75 μm pore size). Finally, placed the cells in 35-mm Petri dishes at a density of approximately 5×10^4^ cells/mL in DMEM supplemented with 10% fetal calf serum (Gibco), 100 U/ml penicillin and 100 mg/ml streptomycin (Gibco) and incubated in a 5% CO_2_ humidified incubator at 37°C. The medium was changed every 2 days, and the explants were examined daily for cell outgrowth using an inverted light microscope. The cells at the third passage were used for the experiments.

Immunostaining of collagen I (1:100 dilution [Abcam, Cambridge, Massachusetts, USA]) and vimentin(1:100 dilution [Abcam, Cambridge, Massachusetts, USA]) was performed to identify the cell type. LF cells of the third passage were treated with different concentrations of recombinant human EGF (0, 1, 10, 20 ng/ml, MedChem Express, Monmouth Junction, NJ) with or without 100 nmol/L erlotinib (MedChem Express, Monmouth Junction, NJ) or 1 ug/ml TGF-β1-neutralizing antibody (anti-TGF-β1, Sigma, Aldrich) for 24 h.

### Identify Cell viability

The cell viability was assessed with the colorimetric reagent, 3-(4,5-dimethylthiazol-2-yl)-2,5-diphenyl tetrazolium bromide (MTT, Solarbio, Beijing, China).LF Cells were seeded into 96-well plates (1x10^4^ cells/well) and incubated at 37°C with 5% CO_2_ for 24 h. After washing the LF cells with PBS solution, 100 µL of DMEM containing 10% MTT solution (5 mg/mL) was added to each well for 4 h. After medium removal, 110 µl of dimethyl sulfoxide (DMSO) was added to each well, and the absorbance of the solution was measured at 490 nm using a multi-mode reader (HTX, Gene, Shanghai, China).

### Western blot Analysis

LF tissues and LF cells were lysed in buffer containing protease inhibitors. Then, the lysate was subjected to sodium dodecyl sulfate polyacrylamide gel electrophoresis and transferred to polyvinylidene difluoride membranes. The membranes were blocked with 5% non-fat milk diluted in Tris-buffered saline for 2 h at room temperature. Next, the membranes were incubated at 4°C overnight with primary antibodies as follows: collagen I (1: 1000, Abcam), collagen III (1: 1000, Abcam), EGF(1: 1000, Abcam), pEGFR (1: 1000, Cell Signaling Technology, Danvers, MA), EGFR (1: 1000, Abcam), TGF-β1(1: 1000, Abcam), pSmad3(1: 1000, Cell Signaling Technology, Danvers, MA), Smad3 (1: 1000, Abcam), and β-actin (1: 1000, ABclonal).Next, the blots were washed and incubated with anti-rabbit or anti-mouse immunoglobulin G peroxidase conjugate (1: 3000, RayBiotech, Beijing, China) for 1.5 h at room temperature. The signals were detected using an enhanced chemiluminescence kit (Beyotime, Shanghai, China).

### Statistical Analysis

Independent sample t-test was used for comparison between the two groups (LSCS group & Non-LSCS group) in terms of age, LF thickness, grade of LF fibrosis and immunohistochemical staining data (IBM SPSS Statistics 22.0, SPSS, Chicago, IL). One-way ANOVA was used to analyze the MTT assay results (GraphPad Prism 5.01, La Jolla, CA). Results are expressed as mean ± SD of all measured parameters. P < 0.05 was considered statistically significant.

## Results

### Ligamentum flavum thickness and fibrosis of the LSCS group and Non-LSCS group

**Fig 1:**
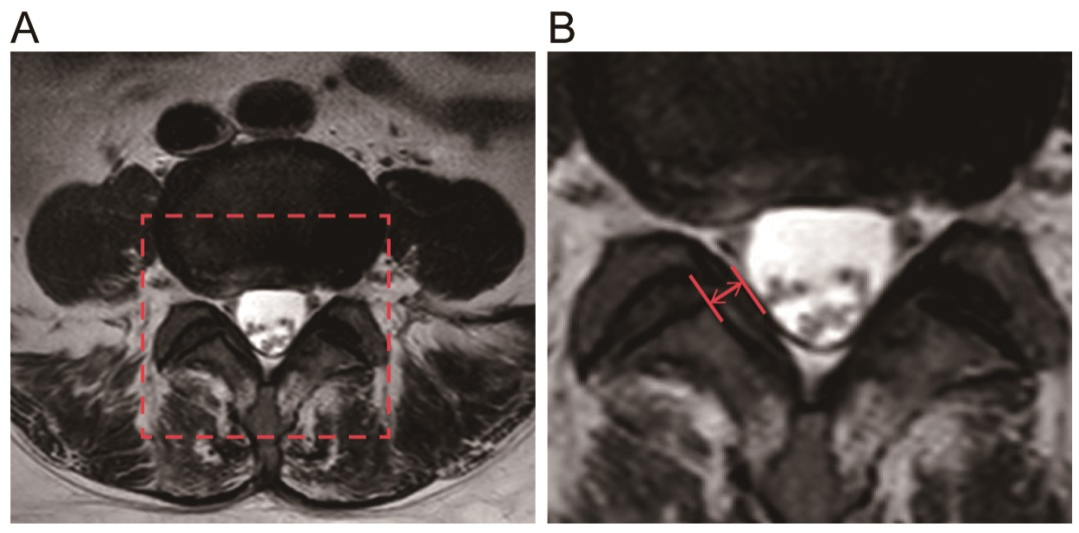
**Measurement of the LF thickness by MRI. A** The red frame was selected as the target analysis area. **B** The red arrow indicates the thickness of the ligamentum flavum at the facet joint level.

**Fig 2:**
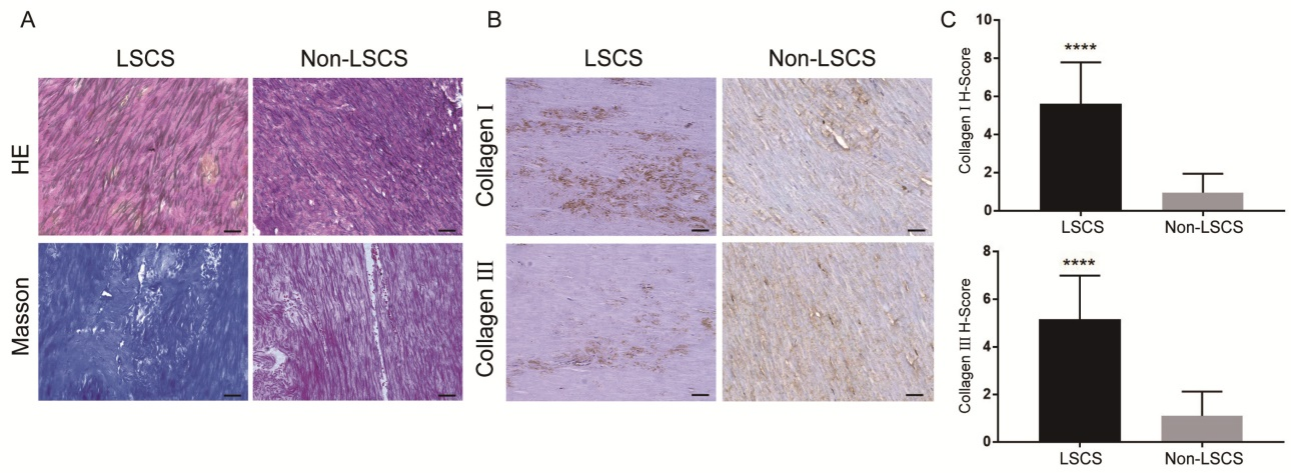
**Morphology and fibrosis of LF tissue in LSCS group and Non-LSCS group. A** H&E staining and Masson trichrome staining of the LSCS group and Non-LSCS group, blue indicates collagen fibers and red indicates elastic fibers. **B** Collagen I and Collagen III immunohistochemical staining of the LSCS group and Non-LSCS group. **C** Statistical analysis of Collagen I and Collagen III levels. The scale bar indicates 50 μm, **** indicates P<0.0001.

The LF thickness of the two patient groups was measured via MRI(Fig. 1), and the results showed that the mean thickness of the LF in the LSCS group(5.81±0.51mm) was significantly higher than that in the Non-LSCS group(2.72±0.45mm) (Table 1). Histological examination of HE staining & Masson trichrome staining (Fig. 2A) showed that compared with the Non-LSCS group, the LSCS group had fewer elastic fibers and more disordered and heterogeneous collagen fibers. This indicated that the LF fibrosis degree was higher in the LSCS group.

### High levels of EGF, pEGFR, TGF-β1, pSmad3, collagen I and collagen III in the LSCS group

**Fig 3:**
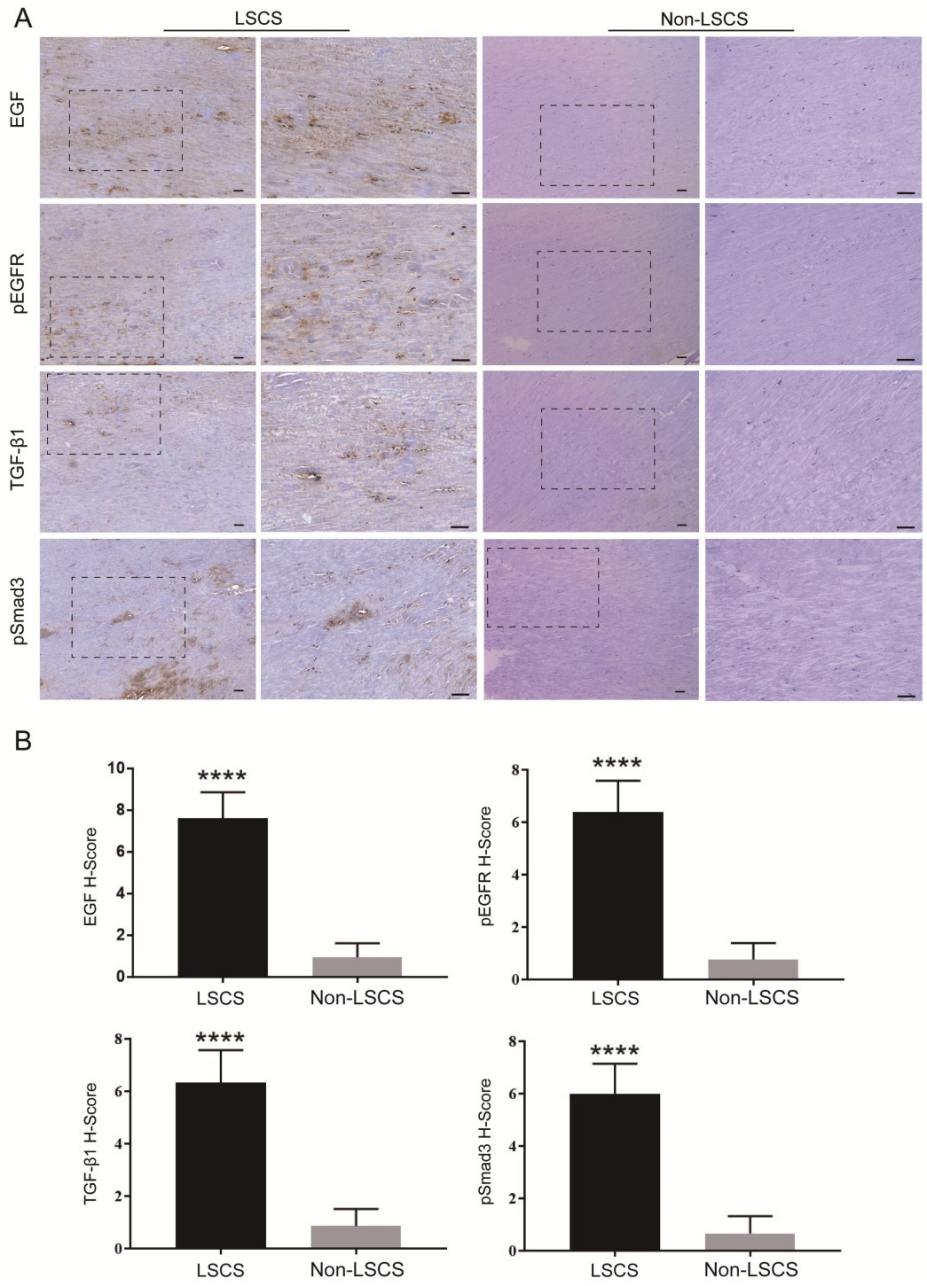
**Immunohistochemical staining of EGF, pEGFR, TGF-β1 and pSmad3 in LF tissue. A** EGF, pEGFR, TGF-β1 and pSmad3 in the LSCS group and Non-LSCS group.** B** Statistical analysis of EGF, pEGFR, TGF-β1 and pSmad3 levels. The scale bar indicates 50 μm, **** indicates P<0.0001.

**Fig 4:**
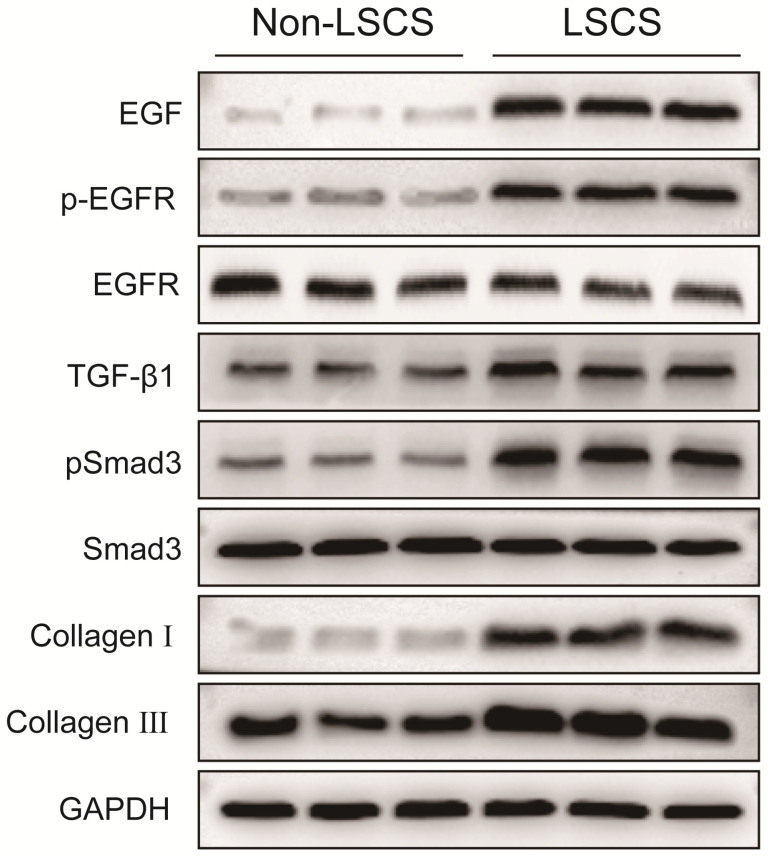
**Protein expression levels of EGF, pEGFR, EGFR, TGF-β1, pSmad3, Smad3, collagen I and collagen III in LF tissue.** The levels of EGF, pEGFR, TGF-β1, pSmad3, collagen I and collagen III in the LSCS group were significantly higher than those in Non-LSCS group. The level of phosphorylated EGFR represents the degree of EGFR activation. When the level of total EGFR is unchanged and the level of phosphorylated EGFR is decreased, it indicates that EGFR activation is decreased or inhibited, and vice versa. Likewise, the level of phosphorylated Smad3 represents the degree of Smad3 activity.

In order to explore the role of EGF signal transduction in ligamentum flavum, immunohistochemical staining and Western blot analysis were performed to analyze the expression of collagen I, collagen III, EGF, pEGFR, TGF-β1 and pSmad3 in the ligamentum flavum of the two groups. The results (Fig. 2B, C) showed that compared with the Non-LSCS group, the expressions of collagen I and collagen III were significantly higher in the LSCS group. Meanwhile, the LSCS group expressed the higher levels of EGF, pEGFR, TGF-β1 and pSmad3 (Fig. 3A, B). Western blot (Fig. 4) analysis showed that the protein expression levels of collagen I, collagen III, EGF, pEGFR, TGF-β1 and pSmad3 in the LSCS group were higher than those in the Non-LSCS group.

### EGF stimulated TGF-β1 activation, collagen I and collagen III expression in LF cells

**Fig 5:**
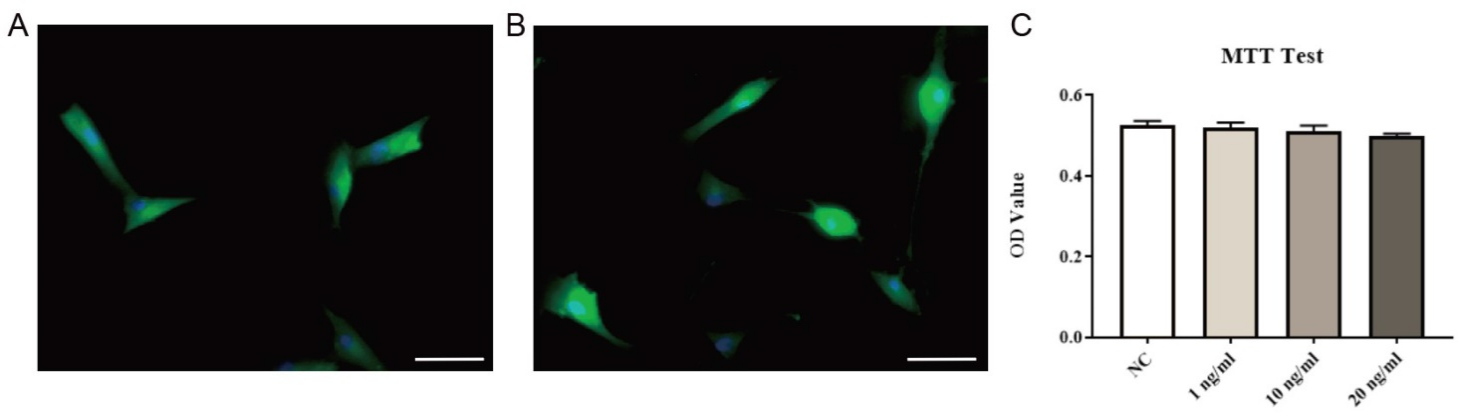
** Identification and viability testing of LF cells.** Immunofluorescence staining of collagen I (**A**) and vimentin (**B**). Ligamentum flavum cells cultured with different concentrations of exogenous EGF were quantitatively analyzed by MTT method, and there was no significant change in cell viability (**C**, P=0.46). Immunofluorescence is shown using FITC (green) and nuclei is shown using DAPI (blue), scale bar indicates 50 μm.

Immunofluorescence staining of primary LF cells extracted and cultured showed that the vast majority of cells were labeled with collagen I or vimentin (markers of LF cells) (Fig. 5A, B), indicating that high-purity LF cells were obtained. Then, after exposing the cells to different concentrations of exogenous EGF, no significant changes in cell viability were found, indicating that exogenous EGF did not show toxicity to LF cells (Fig. 5C).

**Fig 6:**
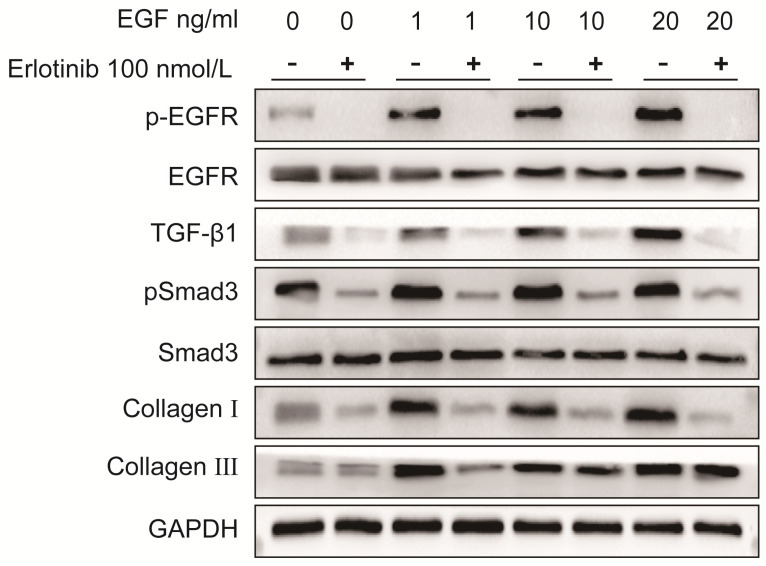
**Protein expression levels of pEGFR, EGFR, TGF-β1, pSmad3, Smad3, collagen I and collagen III in LF cells after exposure to different concentrations of exogenous EGF with or without the EGFR inhibitor erlotinib.** Expression of pEGFR, TGF-β1, pSmad3, collagen I and collagen III in ligamentum flavum cells was significantly increased in a dose-dependent manner after exposed to various concentrations of exogenous EGF without erlotinib, erlotinib significantly attenuated EGF-induced increases in pEGFR, TGF-β1 pSmad3, collagen I and collagen III expression.

**Fig 7:**
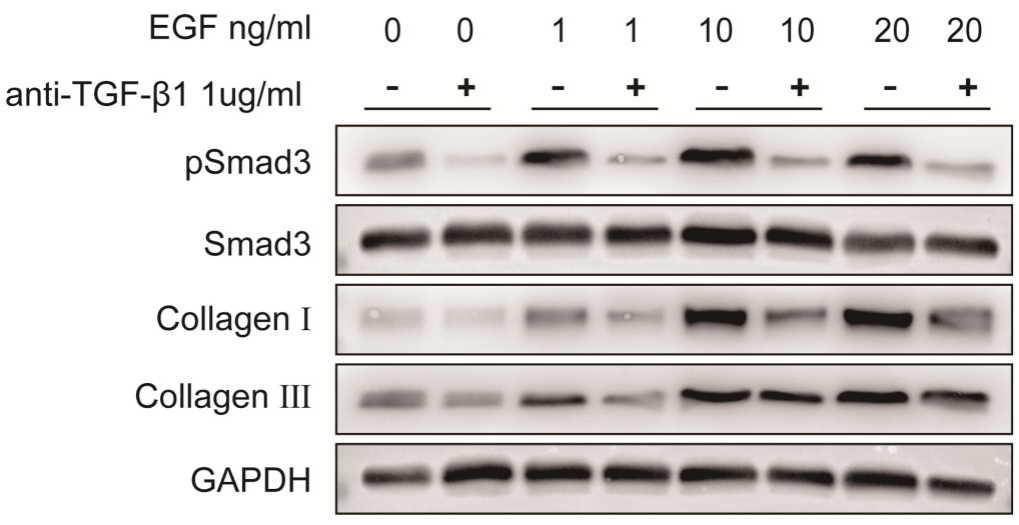
** Protein expression levels of pSmad3, Smad3, collagen I and collagen III in LF cells after exposure to different concentrations of exogenous EGF with or without TGF-β1 neutralizing antibody.** Expression of pSmad3, collagen I and collagen III in ligamentum flavum cells was significantly increased in a dose-dependent manner after exposed to various concentrations of exogenous EGF without TGF-β1 neutralizing antibody, TGF-β1 neutralizing antibody significantly attenuated EGF-induced increases in pSmad3, collagen I and collagen III expression.

As shown in Fig. 6 and Fig. 7, the protein expression levels of pEGFR, TGF-β1, pSmad3, collagen I and collagen III in LF cells were significantly increased in a dose-dependent manner after exposure to exogenous EGF.

### Both erlotinib and TGF-β1 neutralizing antibody can block EGF-induced collagen I and collagen III overexpression in LF cells

To verify the role of EGFR-TGF-β1-Smad3 signaling pathway in LF cells-induced collagen I and collagen III expression, EGFR-specific inhibitor erlotinib and TGF-β1 neutralizing antibody were respectively used to treat LF cells exposed with exogenous EGF. The results showed that EGF-induced expressions of pEGFR, TGF-β1, pSmad3, collagen I and collagen III were significantly decreased in LF cells after treatment with erlotinib (100 nmol/L) (Fig. 6). Likewise, EGF-induced expression of pSmad3, collagen I and collagen III was significantly attenuated in LF cells after treatment with TGF-β1 neutralizing antibody (1 μg/ml) (Fig. 7).

## Discussion

As stated in the Introduction, the dysregulation of EGF expression is associated with a variety of diseases 12-14. In this study, we found for the first time that EGF, pEGFR, collagen I and collagen III expression were upregulated in the LSCS group, by immunohistochemical analysis and western blot analysis of LF tissues. Collagen I and collagen III are important markers of fibrosis and LF hypertrophy. This means that increased EGF expression is associated with fibrosis. Furthermore, western blot analysis of cell experiments showed that exogenous EGF treatment induced a dose-dependent increase in collagen I and collagen III. Most importantly, we found that inhibition of EGF signaling in vitro cell experiments inhibited LF fibrosis, suggesting that targeting EGF signaling may be a novel strategy to prevent and treat LF fibrosis.

The expression of various cytokines was increased in EGF-stimulated LF fibroblasts, including TGF-β1, collagen I and collagen III associated with fibrosis 13. We treated LF fibroblasts with erlotinib, a specific inhibitor of EGFR, to investigate the effect of inhibiting EGFR activity. The expression of collagen I, collagen III and endogenous TGF-β1 was downregulated after treatment of EGF co-cultured fibroblasts with erlotinib. This indicated that erlotinib could significantly inhibit the expression of TGF-β1 in fibroblasts. Erlotinib inhibits the phosphorylation of EGFR in cells and reduces the uptake of EGF by cells, thereby inhibiting the production of TGF-β1. Currently, the underlying mechanism of EGFR activation during ligamentum flavum fibrosis is unclear, but it may be related to persistent stimulation of EGFR ligands. EGF selectively binds to EGFR, undergo autophosphorylation at specific tyrosine residues of EGFR within the intracellular domain, which activate downstream signaling pathways 29. Erlotinib can inhibit the activation of EGFR, inhibit the binding of EGFR and EGF, thereby inhibiting downstream signaling pathways, including the TGFβ1 signaling pathway. It is noteworthy that previous studies have shown that ligamentum flavum hypertrophy is closely related to angiogenesis 30. It is known that EGF itself is an angiogenesis factor, and EGFR activation is usually associated with angiogenesis^31^.Whether erlotinib can inhibit angiogenesis in ligamentum flavum by inhibiting EGFR activity requires further studies to address the relevant cellular and molecular basis.

Since TGF-β1 has been previously found to be an important growth factor, it plays a key role in the fibrosis process in many tissues 17, so the current research focuses on investigating the potential role of TGF-β1 in LF fibrosis and the molecular mechanisms that might be involved 32-34. To date, no studies have revealed the potential functions of EGF and TGF-β1/pSmad3 signaling in ligamentum flavum hypertrophy (LFH). Therefore, this study hypothesized that EGF, through TGF-β1/pSmad3 signaling, may contribute to the pathological process of LFH by activating collagen fiber production. Therefore, to test our hypothesis, we first performed immunohistochemical analysis and western blot analysis of ligamentum flavum tissue. The results all showed that the LSCS group expressed higher levels of TGF-β1, pSmad3, collagen I and collagen III compared to the Non-LSCS group. After further verification, we found that after TGF-β1 neutralizing antibody (TGF-β1-specific inhibitor) treatment of exogenous EGF co-cultured fibroblasts, the expressions of pSmad3, collagen I and collagen III were significantly lower than those in the control group. The above data show that when TGF-β1 is inhibited, the activity of its downstream marker Smad3 is reduced, which in turn leads to a decrease in the secretion of collagen I and collagen III.

The novelty of this study is that we found local high expression of EGF in the ligamentum flavum upregulates the phosphorylation of its receptor (EGFR) and activates the downstream TGF-β1/pSmad3 signaling pathway to enhance collagen I and collagen III production. These results suggest that the EGFR/TGF-β1/Smad3 biological axis can regulate fibrosis in hypertrophic LF, and EGF may be an important target for the treatment of lumbar spinal stenosis caused by ligamentum flavum hypertrophy. Several limitations in this study should be mentioned. First, how EGF and Smad3 are involved in fibrosis in LF has not been investigated. Furthermore, in vivo studies are recommended to validate the effect of EGF on LF hypertrophy.

## Conclusion

In conclusion, in this study, we found high levels of EGF expression and associated signaling pathways in hypertrophic ligamentum flavum. Our study suggests that EGF may contribute to the LFH process by promoting the production of collagen I and collagen III. Furthermore, for the LFH process, the mediating effect of the TGF-β1/Smad3 signaling pathway on EGF-induced collagen I and collagen III overexpression was also validated. Therefore, the current study concludes that EGF contributes to LFH through the TGF-β1/Smad3 signaling pathway. These findings provide a new dimension for understanding the pathogenesis of LFH. Due to the lack of LFH animal models, in the current study, we did not assess whether the inhibition of EGF would attenuate LFH processes in vivo. Therefore, an important component of our further work was to establish LFH animal models and validate the role of EGF in LFH processes in vivo.

## Figures and Tables

**Table 1:** General data of the two groups of patients. LF, Ligamentum flavum; LSCS, lumbar spinal canal stenosis. Independent samples t-test; P < 0.05 is considered to be significant.

	LSCS Group (n=30)	Non-LSCS Group (n=20)	P value
Lumbar level	L4/5	L4/5	
Gender	18 males,12 females	8 males,12 females	
Age (years)	58.35 ± 5.60	55.20 ± 7.75	0.238
LF thickness (mm)	5.81 ± 0.51	2.72 ± 0.45	<0.001
Grade of LF fibrosis	3.2 ± 0.67	0.3 ± 0.18	<0.001
